# Supplementary material for: Impaired LEF1 Activation Accelerates iPSC-Derived Keratinocytes Differentiation in Hutchinson-Gilford Progeria Syndrome
Source: Int J Mol Sci. 2022 May 14;23(10):5499. doi: 10.3390/ijms23105499 (PMC9141373; doi:10.3390/ijms23105499)
Supplement: Supplementary file 1 [file ijms-23-05499-s001.zip › ijms-1683297-supplementary.pdf]

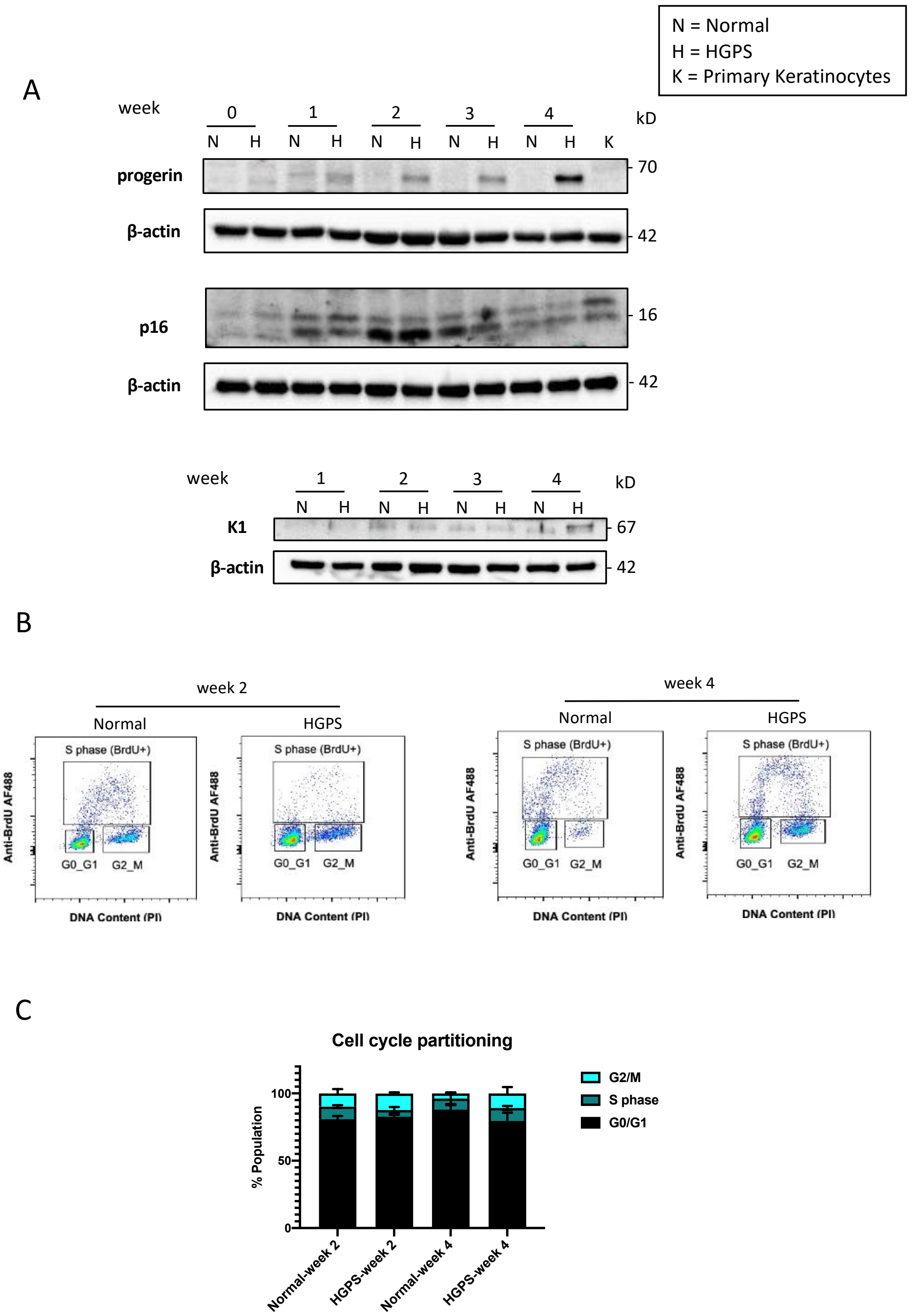

Figure S1. (**A**) Representative Western blot result showing progerin, p16 and K1 expression during normal and HGPS iPSCs-keratinocytes differentiation. (**B**) Representative flow cytometry plots showing BrdU-PI cell cycle analysis during normal and HGPS iPSCs-keratinocytes induction. The distribution of cells at G0/G1, S, and G2/M phases were indicated within each plot. (**C**) Quantification of the cell cycle partitioning during normal and HGPS iPSCs-keratinocytes induction. Data were presented as mean ± SD (n = 3).

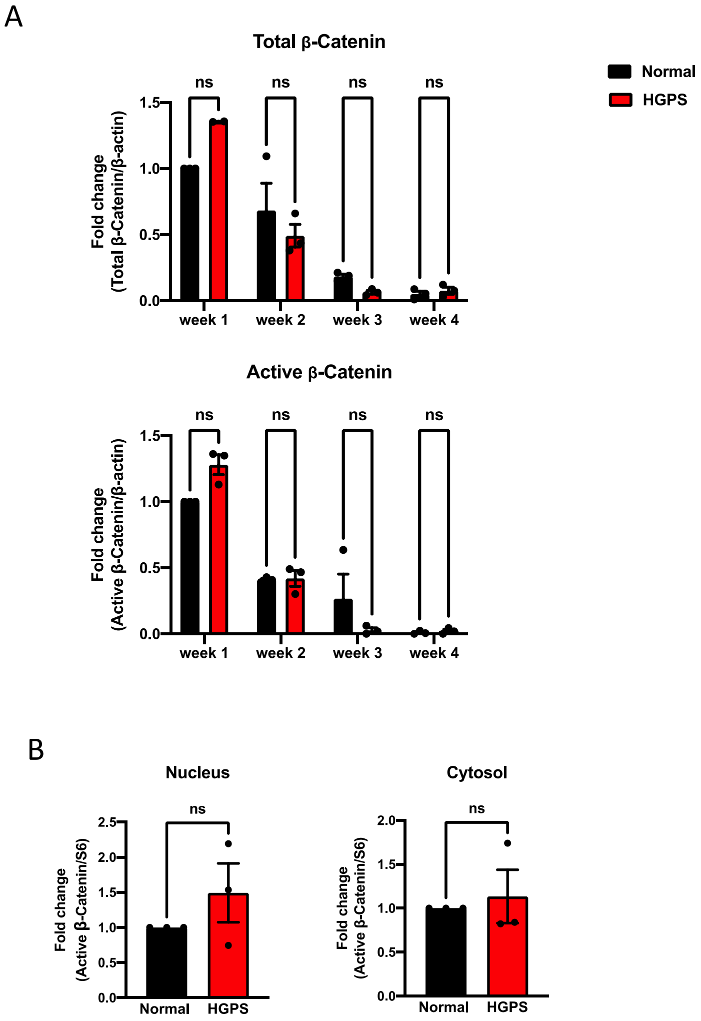

Figure S2. (**A**) Quantification of total and active β-catenin expression during normal and HGPS iPSCs-keratinocytes induction. The Representative Western blot result is shown in Figure 4A. Data were presented as mean ± SD (n = 3). ns, not significant, Two-way ANOVA followed by Sidak's multiple comparisons test. (**B**) Quantification of nuclear and cytosolic active β-catenin expression in early-differentiating (week 1) normal and HGPS iPSCs. The Representative Western blot result is shown in Figure 4B. Data were presented as mean ± SD (n = 3). ns, not significant, unpaired two-tailed t-test.

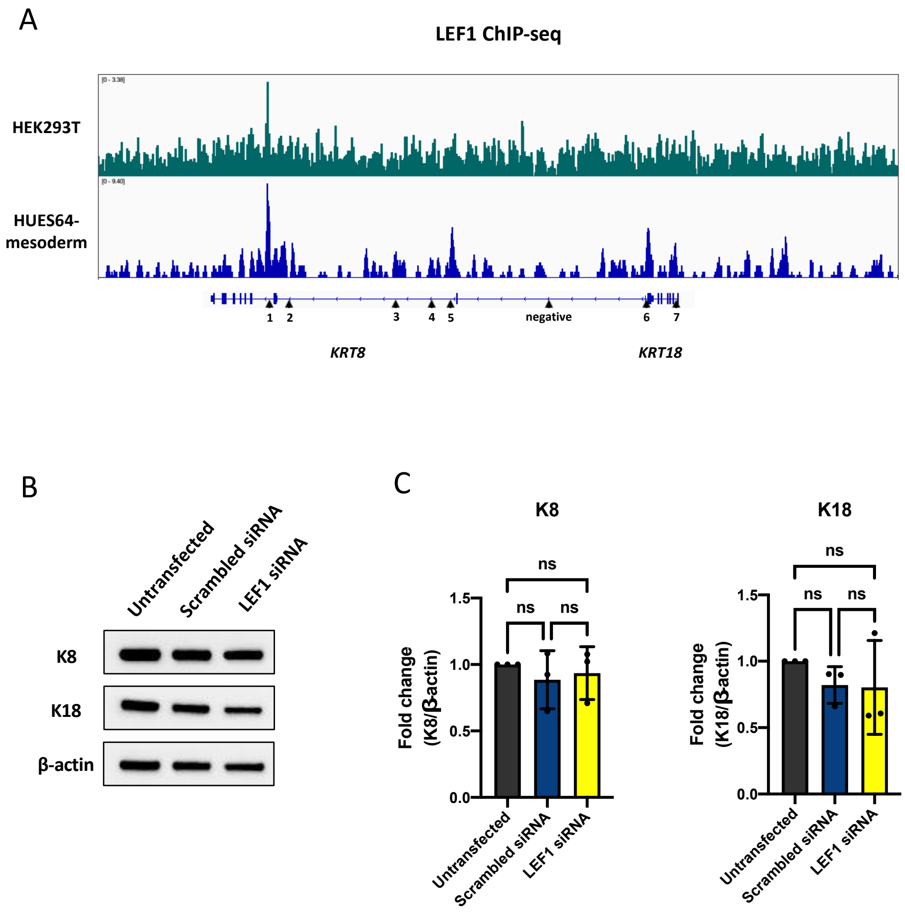

Figure S3. (**A**) Genomic tracks display public available LEF1 ChIP-seq data. Shown are LEF1 enrichment profiles at K8/K18 locus in HEK293T (green) and human embryonic stem cell (HUES64) derived mesoderm (blue). The K8/K18 gene track with putative LEF1 binding sites was shown below the profiles. (**B**) Representative Western blot result showing K8 and K18 protein expression 48 hours after LEF1 siRNA knockdown in normal iPSCs differentiation. (**C**) Quantification of K8 and K18 protein expression after LEF1 siRNA knockdown in (**B**). Data were presented as mean ± SD (n = 3). ns, not significant, One-way ANOVA followed by Tukey’s multiple comparisons test.

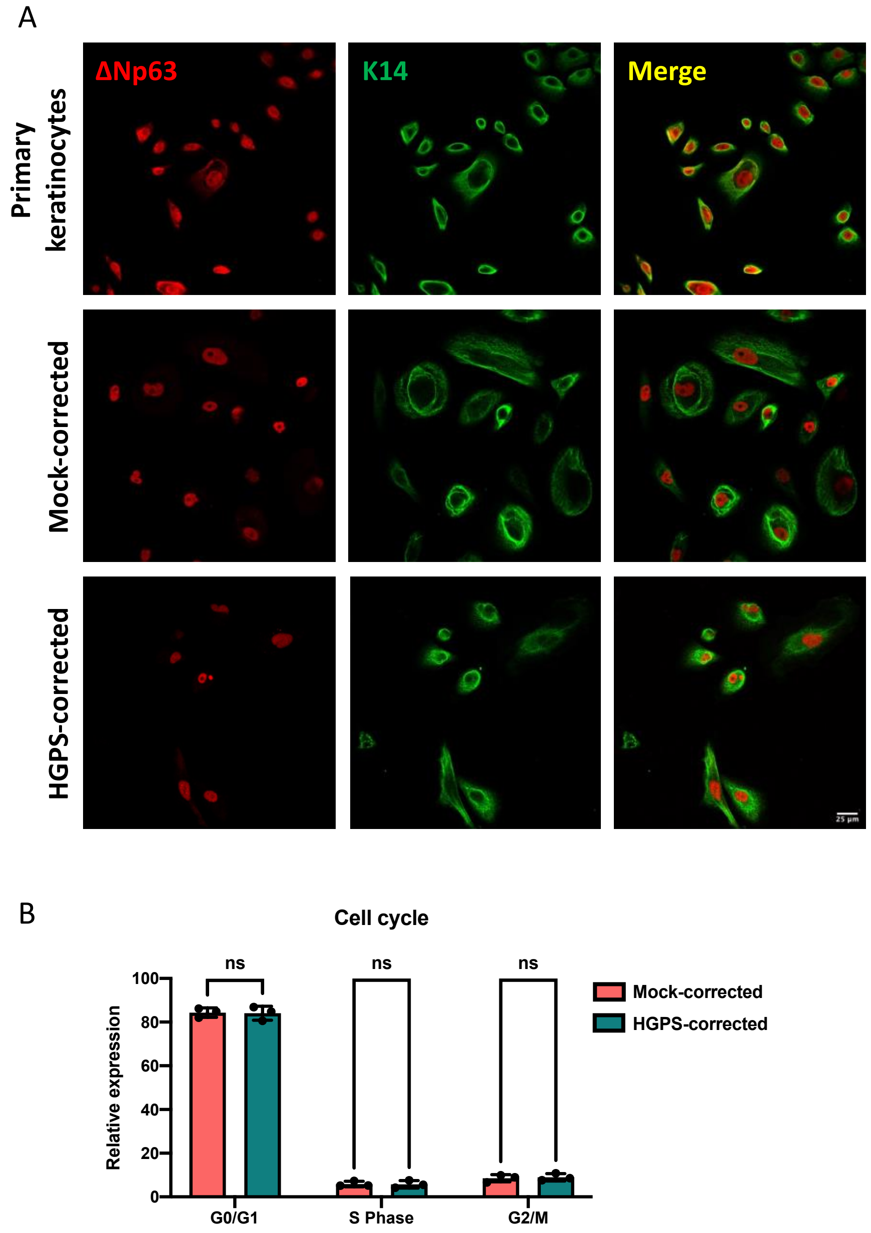

Figure S4. (**A**) Basal layer keratinocyte markers ΔNp63 and K14 expression in mock-corrected and HGPS-corrected iPSCs-derived keratinocytes at week 4 as well as in primary keratinocytes indicated by immunofluorescence staining (scale bar = 25µm). (**B**) Quantification of the cell cycle partitioning in mock-corrected and HGPS-corrected iPSCs-derived keratinocytes at week 4. Data were presented as mean ± SD (n = 3). ns, not significant, Two-way ANOVA followed by Sidak's multiple comparisons test.
